# Dissecting the essentiality of the bifunctional trypanothione synthetase-amidase in *Trypanosoma brucei* using chemical and genetic methods

**DOI:** 10.1111/j.1365-2958.2009.06761.x

**Published:** 2009-06-24

**Authors:** Susan Wyllie, Sandra L Oza, Stephen Patterson, Daniel Spinks, Stephen Thompson, Alan H Fairlamb

**Affiliations:** Division of Biological Chemistry and Drug Discovery, Wellcome Trust Biocentre, College of Life Sciences, University of DundeeDundee, DD1 5EH, Scotland, UK

## Abstract

The bifunctional trypanothione synthetase-amidase (TRYS) comprises two structurally distinct catalytic domains for synthesis and hydrolysis of trypanothione (*N*^1^,*N*^8^-*bis*(glutathionyl)spermidine). This unique dithiol plays a pivotal role in thiol-redox homeostasis and in defence against chemical and oxidative stress in trypanosomatids. A tetracycline-dependent conditional double knockout of TRYS (cDKO) was generated in bloodstream *Trypanosoma brucei*. Culture of cDKO parasites without tetracycline induction resulted in loss of trypanothione and accumulation of glutathione, followed by growth inhibition and cell lysis after 6 days. In the absence of inducer, cDKO cells were unable to infect mice, confirming that this enzyme is essential for virulence *in vivo* as well as *in vitro*. To establish whether both enzymatic functions were essential, an amidase-dead mutant cDKO line was generated. In the presence of inducer, this line showed decreased growth *in vitro* and decreased virulence *in vivo*, indicating that the amidase function is not absolutely required for viability. The druggability of TRYS was assessed using a potent small molecule inhibitor developed in our laboratory. Growth inhibition correlated in rank order cDKO, single KO, wild-type and overexpressing lines and produced the predicted biochemical phenotype. The synthetase function of TRYS is thus unequivocally validated as a drug target by both chemical and genetic methods.

## Introduction

The protozoan parasite *Trypanosoma brucei* is the causative agent of human African trypanosomiasis (HAT), commonly known as sleeping sickness. This disease, thought to be largely controlled in the 1960s, has re-emerged as a major threat to human health due to a number of factors including lack of financial resources, conflict in affected countries and failure to adequately monitor infection. Currently, the World Health Organisation reports that over 400 000 people are infected with HAT, resulting in an annual death toll of more than 50 000 (Stuart *et al*., 2008). In the absence of an effective vaccine, treatment is solely dependent upon a pitifully small repertoire of drugs which suffer from a number of problems including severe toxic side-effects (Fairlamb, 2003) and acquired drug resistance (Barrett and Fairlamb, 1999). To compound these difficulties, many of the current chemotherapeutic treatments also require lengthy periods of hospitalization and are prohibitively expensive (Stuart *et al*., 2008). With no new drugs for the treatment of HAT in the pipeline, a concerted effort is being made to identify, characterize and validate novel molecular targets urgently required for drug discovery.

In the search for anti-parasitic drug targets, metabolic pathways that are both essential for parasite survival and absent from the host are logical starting points (El Sayed *et al*., 2005). One such pathway is the thiol metabolism of trypanosomes and *Leishmania*. These parasites are uniquely dependent upon trypanothione (*N*^1^,*N*^8^-bis(glutathionyl)spermidine, T[SH]_2_) as their principal thiol, in contrast to most other organisms (including their mammalian hosts) which utilize glutathione (γ-l-glutamyl-l-cysteinylglycine, GSH) (Fairlamb *et al*., 1985). This dithiol is primarily responsible for the maintenance of thiol-redox homeostasis within trypanosomatids, and is involved in a number of critical cellular processes including deoxyribonucleotide synthesis (Dormeyer *et al*., 2001), and defence against oxidative stress (Ariyanayagam and Fairlamb, 2001) and xenobiotics (Vickers *et al*., 2004). Trypanothione has also been implicated in the mode of action as well as the mechanism of resistance of antimonial drugs in *Leishmania* spp. (Mukhopadhyay *et al*., 1996; Wyllie *et al*., 2004; Croft *et al*., 2006) and in defence against chemical and oxidant stress induced by arsenicals and nifurtimox in *T. brucei* (Fairlamb *et al*., 1989; Ariyanayagam *et al*., 2005; Alibu *et al*., 2006). Therefore, in an attempt to exploit this essential and unique metabolite, T[SH]_2_-dependent and biosynthetic enzymes have become a major focus for drug discovery.

The pivotal role of T[SH]_2_ metabolism in the viability and virulence of trypanosomatids has been unequivocally demonstrated using genetic techniques such as classical gene knockout and RNA interference (RNAi). Trypanothione-dependent enzymes that have been validated as potential drug targets using these methodologies include: trypanothione reductase (Tovar *et al*., 1998; Krieger *et al*., 2000), tryparedoxin (Wilkinson *et al*., 2003) and tryparedoxin peroxidase (Wilkinson *et al*., 2003). Unsurprisingly, the enzyme responsible for the biosynthesis of T[SH]_2_, the bifunctional trypanothione synthetase-amidase (TRYS), has also been confirmed as essential for the viability and proliferation of *T. brucei* bloodstream (Comini *et al*., 2004) and procyclic forms (Ariyanayagam *et al*., 2005) by RNAi. In pathogenic trypanosomatids, TRYS catalyses the synthesis of T[SH]_2_ from GSH and spermidine in a two-step, ATP-dependent reaction via a glutathionylspermidine intermediate. In addition to this role in biosynthesis, the N-terminal domain of TRYS also functions as an amidase, hydrolysing T[SH]_2_ to GSH and spermidine, again via glutathionylspermidine. The biological significance of TRYS-amidase, classified as a cysteine–histidine-dependent aminohydrolase/peptidase amidase (Bateman and Rawlings, 2003; Rigden *et al*., 2003), is not fully understood. However, it has been suggested that the conflicting biosynthetic and hydrolytic functions of this enzyme may enable the parasite to respond to fluctuating cellular requirements for both T[SH]_2_ and polyamines, essential for proliferation and differentiation (Fairlamb and Cerami, 1992; Bacchi and Yarlett, 1995).

In the current study, we assessed the essentiality of TRYS by classical gene replacement to fulfil the stringent target validation criteria of the University of Dundee Drug Discovery Unit (Frearson *et al*., 2007). Gene replacement is the preferred method of genetic validation because ‘off-target’ effects cannot be ruled out using RNAi. Moreover, target knock-down by RNAi does not permit independent analysis of bifunctional enzymes such as DHFR-TS (Sienkiewicz *et al*., 2008) or TRYS. Using a combination of classical gene replacement, mutagenesis and chemical intervention methods, we dissect the opposing synthetic and hydrolytic functions of TRYS in order to independently study their respective roles in *T. brucei* cell virulence and viability.

## Results and discussion

### Generation of TRYS conditional null mutants

**Fig. 1:**
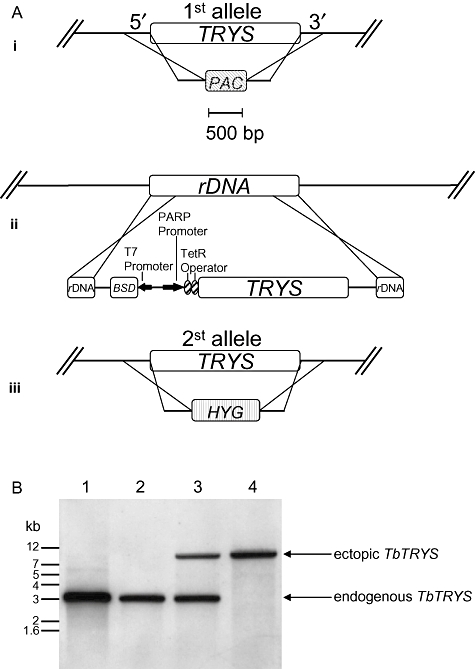
Genotypic analysis of WT, SKO and cDKO cell lines. A. Schematic representation of the stepwise generation of the TRYS cDKO cell line in *T. brucei*. (i) One allele of *TRYS* was replaced with the puromycin-resistance gene (PAC) by homologous recombination, generating Δ*TRYS*::PAC cell line (SKO); (ii) a tetracycline-inducible ectopic copy of *TRYS* was introduced into the rDNA, generating *TRYS*^Ti^Δ*TRYS*::PAC cell line; (iii) while tetracycline induces the expression of the ectopic copy, the remaining allele was replaced with a hygromycin resistance gene (HYG) by homologous recombination, resulting in conditional double knockout cell line *TRYS*^Ti^Δ*TRYS*::PAC/Δ*TRYS*::HYG (cDKO). B. Confirmation of genotype of *T. brucei* TRYS conditional double knockout cell line. Southern blot analysis of PstI-digested genomic DNA (∼5 μg) from wild-type *T. brucei* cells (lane 1), ΔTRYS::PAC (lane 2), *TRYS*^Ti^Δ*TRYS*::PAC (lane 3) and *TRYS*^Ti^Δ*TRYS*::PAC/Δ*TRYS*::HYG (lane 4) cells; the *TRYS* ORF probe shows allelic *TbTRYS* at 3 kb and the ectopic copy *TbTRYS*^Ti^ at ∼10 kb.

To date, the essentiality of *T. brucei* trypanothione synthetase (TRYS) has only been demonstrated by RNAi (Comini *et al*., 2004; Ariyanayagam *et al*., 2005), a methodology that lacks the robustness of classical gene replacement. With this in mind, we sought to definitively classify this enzyme as a drug target in bloodstream trypanosomes by replacing *TRYS* with drug-resistance genes (Fig. 1A). Previous studies have demonstrated that *TRYS* is present as a single-copy gene per haploid genome of *T. brucei* (Oza *et al*., 2003). The first *TRYS* gene copy was replaced with the puromycin *N*-acetyl transferase (*PAC*) gene by homologous recombination and subsequent selection for puromycin resistance, generating a single knockout (SKO) cell line. Attempts to create a null mutant by directly replacing the second allelic copy with the hygromycin-resistance gene (hygromycin phosphotransferase, *HYG*) proved unsuccessful, suggesting that TRYS is indeed essential for the viability of *T. brucei* bloodstream parasites. Consequently, a conditional double knockout (cDKO) cell line was generated by introducing an ectopic and tetracycline-inducible copy of *TRYS* prior to replacing the second copy with *HYG*. This ectopic copy of *TRYS* was inserted into the rDNA locus of the SKO cell line using a pLew 100 vector encoding a blasticidin-resistance gene (*BSD*) followed by deletion of the second genomic copy with HYG. As the modified *T. brucei* 427 cell line [wild-type (WT)] used in this study constitutively expresses the T7 RNA polymerase and the tetracycline repressor protein, the resulting cell line was a *TRYS* conditional null mutant where TRYS expression depends on the presence of tetracycline (cDKO). Southern blot analysis of genomic DNA from cell lines generated at each stage of this process confirmed the validity of the *TRYS* conditional null mutant (Fig. 1B).

### TRYS is essential in bloodstream *T. brucei in vitro*

**Fig. 2:**
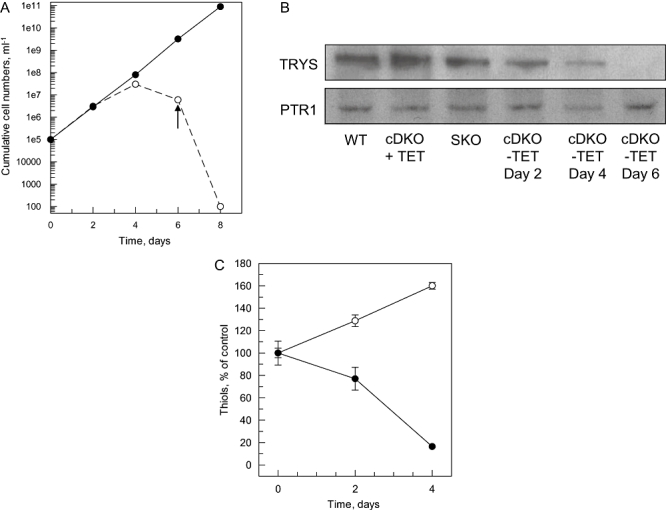
Growth characteristics and biochemical analysis of cDKO cells *in vitro*. A. The growth of the cDKO cell line in HMI9-T media was monitored in the presence (closed circles) and absence of tetracycline (open circles, dashed line). The arrow indicates the point where the viable cells are no longer recoverable when subcultured for up to 10 days in medium containing tetracycline. B. Immunoblots of cell extracts of WT, SKO and cDKO (plus and minus tetracycline) cells were probed with antiserum to *T. brucei* TRYS and to *T. brucei* PTR1 as a control (1 × 10^7^ parasites in each lane). C. Intracellular T[SH]_2_ (closed circles) and GSH (open circles) levels in cDKO cells following the removal of tetracycline from cultures. Initial levels of T[SH]_2_ and GSH in untreated cells were 0.42 and 0.54 nmol(10^8^ cells)^−1^ respectively. Each data point represents the means ± standard deviations from triplicate determinations.

In HMI9-T culture medium, no significant difference could be detected between the growth rates of the WT and SKO cells, with generation times of 9.6 ± 0.1 and 9.5 ± 0.2 h respectively (data not shown). Indeed, the cDKO cell line, grown in the presence of tetracycline, showed no marked growth phenotype with a doubling time of 9.9 ± 0.02 h (Fig. 2A). Following the removal of tetracycline from the medium, cDKO cells continued to grow exponentially for 3 days. Growth slowed by day 4 of culture with all cells finally dying by day 8. To determine the actual point where cDKO parasites grown in the absence of tetracycline completely lose viability, cells were subcultured each day into medium containing tetracycline. Up to the 6th day of culture cells remained viable in the absence of tetracycline; however, beyond this point no viable cDKO cells could be recovered on culture for a further 10 days. Failure of this cell line to grow in the absence of the tetracycline-induced expression of TRYS confirms that this enzyme is essential for bloodstream *T. brucei* viability. Interestingly, ectopic expression of the *L. major* TRYS was equally capable of complementing for the loss of endogenous *TRYS* in the cDKO cell line (Fig. S1). The fact that loss of TRYS activity is trypanocidal rather than cytostatic is highly advantageous from a drug discovery perspective because drug therapy is not dependent on a fully functional immune response (Frearson *et al*., 2007).

### Biochemical analyses of TRYS cDKO cells

The ‘slow death’ phenotype of cDKO cells following the removal of tetracycline may be partly explained by the low turnover of TRYS or its product, T[SH]_2_. Western blot analysis of whole cell extracts revealed that although the levels of this enzyme declined following the removal of tetracycline, it was not until day 6 that TRYS was no longer detectable (Fig. 2B). This observation suggests that the rate of turnover of TRYS (or T[SH]_2_) is very low in *T. brucei* and that TRYS (or T[SH]_2_) is only removed from the cell by dilution due to cell division in the absence of further protein synthesis. Nevertheless, the death of cDKO cells coinciding with the disappearance of TRYS once again confirms that this enzyme is essential in bloodstream trypanosomes.

The effect of TRYS depletion on intracellular thiols was studied by high-performance liquid chromatography (HPLC). Due to the number of cells required for this analysis, thiols could only be monitored in cultures for 4 days following the removal of tetracycline. The cessation of ectopic TRYS expression within these parasites had a pronounced effect on intracellular thiol levels (Fig. 2C). Glutathione, the substrate of TRYS, accumulated in cDKO cells in the absence of tetracycline, such that after 4 days, levels had reach 160% of those seen in control cells (cDKO cells plus tetracycline). In contrast, T[SH]_2_ and glutathionylspermidine, the products of this enzyme reaction, fell considerably. Indeed, T[SH]_2_ levels within these parasites fell to 16.5% of control levels. As 4 day cultures showed only minimally retarded growth in comparison with control cells, it would appear that bloodstream trypanosomes, at least *in vitro*, can survive with vanishingly small amounts of T[SH]_2_. However, it should be noted that the HMI9-T culture medium used in these studies is rich in reducing agents such as thioglycerol (56 μM) and cysteine (1.5 mM) which may protect these thiol-depleted cells from oxidant stress.

### Virulence of cDKO parasites in mice

**Fig. 3:**
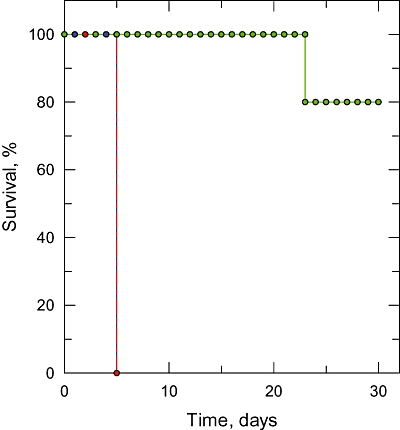
Virulence of cDKO parasites in mice. Groups of five mice were infected with either WT or cDKO cells (1 × 10^4^ parasites). Mice infected with tetracycline-treated parasites were dosed with doxycycline in their drinking water 3 days prior to, and throughout, the infection. Data are presented in the form of a Kaplan–Meier survival plot. Symbols: WT, blue; cDKO in the presence of doxycycline, red; cDKO in absence of doxycycline, green.

The oxidative environment *in vivo* is significantly different from *in vitro* culture conditions, underlining the importance of carrying out drug target validation studies in appropriate animal models (Frearson *et al*., 2007). With this in mind, groups of mice were inoculated with WT, cDKO or cDKO parasites which had been grown in the absence of tetracycline for 24 h. Mice infected with tetracycline-treated parasites were dosed with doxycycline in their drinking water 5 days prior to, and throughout infection to maintain expression of ectopic TRYS. Infections were monitored over a 30 day period and survival curves of each infection are shown in Fig. 3. Mice infected with WT parasites succumbed to infection on day 5 and this was also the case for mice infected with cells expressing ectopic TRYS (cDKO plus doxycycline). In contrast, four out of five mice infected with cDKO cells in the absence of doxycycline remained completely free of parasites beyond 30 days while the remaining mouse from this group only showed a lethal parasitaemia on day 23. Western blot analysis of cells recovered from this infected mouse confirmed that these parasites had regained expression of TRYS, suggesting that tetracycline control had been lost, a common phenomenon in conditional null mutants of essential genes in *T. brucei* (Chang *et al*., 2002; Roper *et al*., 2002). Collectively, these results confirm that TRYS is essential for parasite survival in a mammalian host.

### Chemical validation of TRYS as a drug target in *T. brucei*

**Fig. 4:**
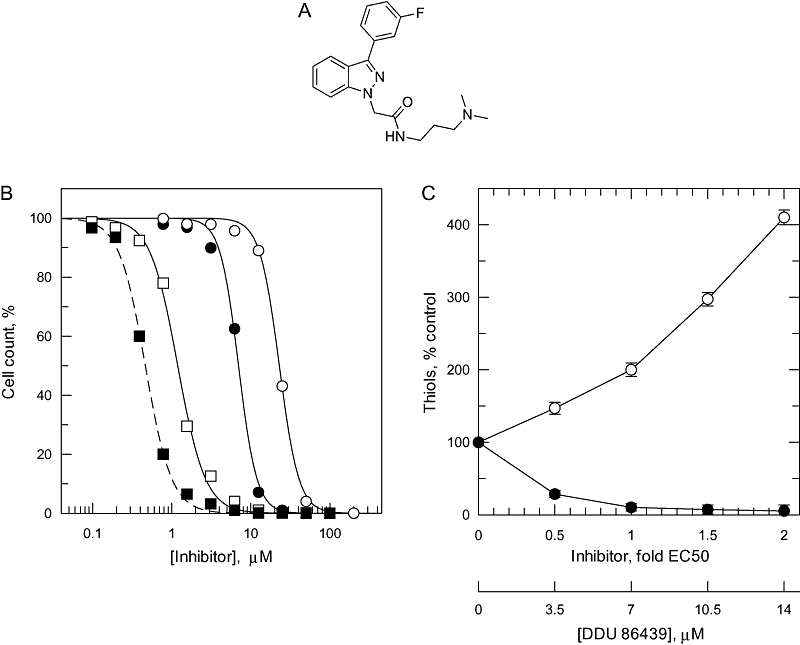
Chemical targeting of TRYS in *T. brucei*. A. Chemical structure of TRYS inhibitor DDU 86439 (*N-*(3-(dimethylamino)propyl)-2-(3-(3-fluorophenyl)-1*H*-indazol-1-yl)acetamide). B. EC_50_ values were determined for DDU 86439 against WT (closed circles), SKO (open squares), TRYS-overexpressing (open circles) and cDKO cells grown in the absence of tetracycline for 2 days prior to analysis (closed squares, dotted line). The curves are the non-linear fits of data using a two-parameter EC_50_ equation provided by GraFit (see *Experimental procedures*). EC_50_ values of 7.1 ± 0.2, 1.2 ± 0.05, 23.3 ± 0.3 and 0.46 ± 0.01 μM were determined for WT, SKO, TRYS-overexpressing and cDKO cell lines respectively. Data are the mean of duplicate measurements. C. Analysis of intracellular T[SH]_2_ (closed circles) and GSH (open circles) levels in *T. brucei* bloodstream parasites (WT) following incubation with compound DDU 86439 (72 h). Data are represented as a percentage of the thiol levels of untreated cells (0.51 and 0.42 nmol(10^8^ cells)^−1^ for GSH and T[SH]_2_ respectively; Table S1) and each measurement is the mean of three individual measurements.

Genetic validation is a crucial first step in the identification of a drug target. However, the importance of demonstrating that a target is ‘druggable’ within a cell should not be underestimated. As a result of a high-throughput drug screening and medicinal chemistry campaign carried out by the Drug Discovery Unit in Dundee, DDU 86439 (*N-*(3-(dimethylamino)propyl)-2-(3-(3-fluorophenyl)-1*H*-indazol-1-yl)acetamide, Fig. 4A) was identified as a potent inhibitor of recombinant *T. brucei* TRYS with an IC_50_ of 45 nM (full details are to be published elsewhere). This compound inhibited the growth of WT cells in HMI9-T media with an EC_50_ value of 7.0 ± 0.2 μm following a 72 h exposure. To confirm that TRYS was specifically targeted by this compound, TRYS SKO and overexpressing (OE) cell lines were compared with WT cells for their relative sensitivity to compound DDU 86439. Western analysis and densitometry demonstrated that TRYS protein levels were approximately twofold lower in SKO cells (Fig. 2B) and threefold higher in OE cells as compared with WT levels (Fig. S2). Changes in the level of TRYS in these cells correlated well with their relative sensitivity to compound DDU 86439 with EC_50_ values of 1.2 ± 0.04 and 23.0 ± 0.4 μM for SKO and OE cell lines respectively (Fig. 4B). cDKO cells, grown in the absence of tetracycline for 2 days prior to analysis, were found to be hypersensitive to this compound (EC_50_ 0.46 ± 0.01 μM, Fig. 4B), providing further evidence that TRYS is the specific target of this inhibitor.

Intracellular thiol levels were determined in WT parasites following incubation with concentrations of the TRYS inhibitor corresponding to 0.5, 1.0, 1.5 and 2.0 times the established EC_50_ value (7 μM) (Fig. 4C). Treatment with DDU 86439 resulted in a significant, dose-dependent decrease in the levels of intracellular T[SH]_2_. Trypanosomes incubated with the highest levels of this compound (14 μM) were found to maintain only 5% of their original T[SH]_2_ levels after 72 h. As in the case of cDKO cells (Fig. 2B), the absence of functional TRYS in drug-treated cells led to an accumulation of GSH, with levels reaching fourfold higher than those seen in untreated cells. The greater speed and magnitude of these effects are consistent with the acute effects of chemical inhibition compared with the slower effects of loss of target activity by genetic intervention (Fig. 2D). These observations also suggest that the minimum concentration of T[SH]_2_ compatible with survival is ∼3 μM [Table S3, assuming 10^8^ cells = 5.8 μl (Opperdoes *et al*., 1984)].

### Is the amidase activity of TRYS essential to bloodstream *T. brucei*?

Following the unequivocal chemical and genetic validation of the synthetase activity of TRYS as a drug target in bloodstream *T. brucei*, we posed the question: could this bifunctional enzyme actually embody two drug targets in one? Comparatively little is known about the amidase activity of TRYS and its biological function remains unclear. In previous kinetic and structural studies, a cysteine at position 59 of the equivalent *Crithidia fasiculata* (Oza *et al*., 2002a) and *L. major* (Fyfe *et al*., 2008) enzymes has been demonstrated as critical in the catalytic mechanism of the TRYS-amidase. Replacement of cysteine 59 with an alanine in the *Crithidia* enzyme resulted in a functional synthetase devoid of amidase activity. To determine the role of TRYS-amidase in parasite viability and virulence, this knowledge was exploited to generate a TRYS conditional null mutant cell line (cDKO(C57A)) expressing an amidase-‘dead’ enzyme under tetracycline control (Fig. S3).

**Fig. 5:**
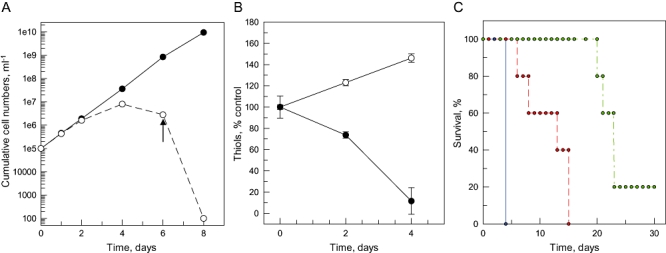
*In vitro* and *in vivo* characterization of a TRYS-amidase ‘dead’ cell line. A. The growth of the cDKO(C57A) cell line in HMI9-T medium was monitored in the presence (closed circles) and absence of tetracycline (open circles, dashed line).The arrow indicates the point where the viable cells are no longer recoverable when subcultured for up to 10 days in medium containing tetracycline. B. Intracellular T[SH]_2_ (closed circles) and GSH (open circles) levels in cDKO(C57A) cells following the removal of tetracycline from cultures. Each data point represents the means ± standard deviations from triplicate determinations. C. Groups of five mice were infected with either WT or cDKO(C57A) cells (1 × 10^4^ parasites). Mice infected with tetracycline-treated parasites were dosed with doxycycline in their drinking water 3 days prior to, and throughout, the infection. Data are presented in the form of a Kaplan–Meier survival plot. Symbols: WT, blue; cDKO in the presence of doxycycline, red; cDKO in absence of doxycycline, green.

The amidase dead cell line was viable in HMI9-T medium in the presence of tetracycline with a generation time of 11.1 ± 0.1 h (Fig. 5A), marginally slower than the original cDKO line (9.9 ± 0.02 h). Removal of tetracycline again resulted in the slow death of parasites over an 8 day period with viability being lost on day 6 of culture, confirming that TRYS synthetase, but not amidase activity is essential for growth *in vitro*. In an identical manner to the cDKO cell line, intracellular T[SH]_2_ levels fell following the removal of tetracycline from cDKO(C57A) cultures while GSH steadily accumulated (Fig. 5B). Parasites expressing the amidase-dead TRYS were markedly less virulent in mice than those expressing the functional enzyme (Fig. 5C). All five mice infected with cDKO(C57A) gradually succumbed to infection over a 9 day period, from day 6–15, while cDKO-infected animals died on day 5. The parasites recovered from cDKO(C57A)-infected mice continued to express the mutated form of the TRYS enzyme, as determined by PCR sequence analysis of genomic DNA (data not shown). Four out of five mice infected with cDKO(C57A) cells in the absence of doxycycline remained free of parasites for more than 20 days before becoming terminally infected, while one mouse remained parasite-free for the entire 30 days of the experiment. Western and PCR analysis of parasites recovered from the infected animals revealed that tetracycline control had once again been lost and the mutated TRYS was being freely expressed (data not shown). While it is evident that TRYS-amidase activity is not essential for the *in vivo* or *in vitro* viability of *T. brucei*, the absence of this activity retards growth *in vitro* and decreases virulence in the mouse model.

### What is the biological function of TRYS-amidase in *T. brucei*?

**Fig. 6:**
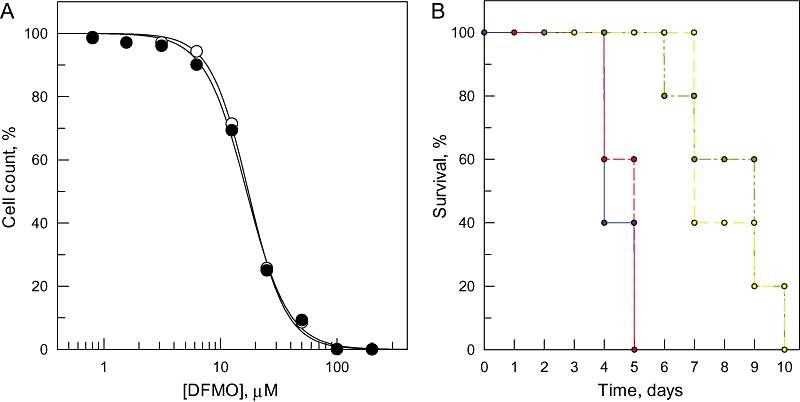
The effect of polyamines on the growth and virulence of cDKO(C57A) parasites. A. EC_50_ values were determined for DFMO against cDKO (closed circles) and cDKO(C57A) cells (closed circles). The curves are the non-linear fits of data using a two-parameter EC_50_ equation provided by GraFit (see *Experimental procedures*). EC_50_ values of 17.5 ± 0.5 and 16.6 ± 0.8 μM were determined for cDKO and cDKO(C57A) cell lines respectively. Data are the mean of duplicate measurements. B. Groups of five mice were infected with either cDKO or cDKO(C57A) cells (1 × 10^4^ parasites). All mice were infected with tetracycline-treated parasites and dosed with doxycycline in their drinking water. One set of the cDKO and cDKO(C57A)-infected mice received daily intraperitoneal injections of spermidine (100 mg kg^−1^). Data are presented in the form of a Kaplan–Meier survival plot. Symbols: cDKO, blue; cDKO plus spermidine, red; cDKO(C57A), green; cDKO(C57A) plus spermidine, yellow.

As TRYS-amidase is not required for the viability or virulence of bloodstream *T. brucei*, the question of its biological function remains. Several researchers have suggested that the ability to hydrolyse T[SH]_2_ to its components, GSH and spermidine may enable cells to respond to conditions of polyamine deprivation (Oza *et al*., 2002b; Fyfe *et al*., 2008). To examine this hypothesis, cDKO and cDKO(C57A) cell lines were tested for their relative sensitivity to difluoromethylornithine (DFMO) (Fig. 6A), a suicide inhibitor of ornithine decarboxylase, the rate-determining enzyme in polyamine biosynthesis (Phillips and Wang, 1987; Phillips *et al*., 1988). Should the primary role of TRYS-amidase be to release polyamine stores from T[SH]_2_ in times of need, it would be reasonable to assume that cells without this ability would be more sensitive to DFMO. However, the cell line expressing the functional TRYS-amidase was just as sensitive to DFMO as the cell line expressing the ‘dead’ enzyme (EC_50_: 17.5 ± 0.5 and 16.6 ± 0.8 μM respectively).

To determine whether the reduced virulence of the cDKO(C57A) cell line could be attributed to an inability to access T[SH]_2_-conjugated polyamines, groups of cDKO(C57A)-infected mice were given a daily bolus of spermidine via intraperitoneal injection. Previous studies have shown that dosing of mice in this manner increased parasite polyamine levels to such an extent that DFMO could no longer cure infection (McCann *et al*., 1981; Nathan *et al*., 1981). Similarly, we hypothesized that increased polyamine levels in cDKO(C57A) cells may result in a return to the levels of virulence seen in cDKO infections. Mice inoculated with the cDKO cell line succumbed to infection on days 4 and 5, regardless of the presence or absence of additional spermidine (Fig. 6B). Once again, the cDKO(C57A) parasites were less virulent than those expressing a functional TRYS-amidase, with mice becoming heavily infected between days 6 and 10. Most significantly, daily dosing of infected mice with spermidine had absolutely no effect on the virulence of the cDKO(C57A) cells. The failure of additional spermidine to enhance the virulence of these cells could suggest that the primary function of TRYS-amidase is not to enable access to T[SH]_2_-conjugated polyamines.

These findings bring into question the hypothesis that TRYS-amidase plays a pivotal role in the regulation of cellular polyamines (Shim and Fairlamb, 1988; Tetaud *et al*., 1998; Oza *et al*., 2002b; Fyfe *et al*., 2008). If this is not the case, then what is the biological function of the amidase in *T. brucei*? Kinetic analysis of recombinant *T. brucei* and *T. cruzi* TRYS has shown that the amidase activity of these enzymes is equivalent to less than 1% of the synthetase activity under optimal assay conditions (Oza *et al*., 2002b; 2003). Should this also be true of the *in vivo* enzyme, it is difficult to see how the amidase could have any meaningful impact on intracellular polyamine levels. Alternatively, the low enzymatic activity of recombinant TRYS-amidase could be readily explained if T[SH]_2_ and GspdSH were not the natural substrates of this enzyme. In the Gram-positive bacteria *Mycobacterium smegmatis*, a thiol *S*-conjugate amidase has been characterized which specifically hydrolyses thiol-conjugated xenobiotics, resulting in detoxification of the xenobiotic and recycling of the thiol (Newton *et al*., 2000). It is tempting to suggest that TRYS-amidase may play a similar role in *T. brucei* and that this enzyme is highly selective for *S-*conjugates of T[SH]_2_ formed by trypanothione *S*-transferase (Vickers and Fairlamb, 2004). Studies are underway to test the validity of this hypothesis. What is irrefutable from this study is that TRYS-amidase is not a viable drug target in the African trypanosome.

### Implications for parasite chemotherapy

In this study, TRYS has been chemically and genetically validated as a drug target in bloodstream *T. brucei*. We have shown that TRYS null trypanosomes are unable to establish an infection in mice and that chemical targeting of this enzyme leads to parasite death. The potency of our lead TRYS inhibitor against bloodstream trypanosomes (EC_50_ 7 μM) compares favourably with that of eflornithine (EC_50_ 17.5 μM, Fig. 6A), a current front-line drug used in the treatment of HAT. Like eflornithine, DDU 86439 is not rapidly cidal and the decrease of intracellular T[SH]_2_ content is compatible with dilution due to cell division rather than high metabolic turnover. Given that many existing drugs interact directly or indirectly with T[SH]_2_ metabolism (Fairlamb, 2003), inhibitors of TRYS could prove ideal candidates for combination therapy. RNAi studies have revealed that TRYS-depleted *T. brucei* procyclics are significantly more susceptible to trypanocides, such as melarsoprol and triostam, also known to act on T[SH]_2_ metabolism (Ariyanayagam *et al*., 2005). In addition, these procyclics were also more sensitive to the redox-cycling drug nifurtimox, currently in phase III clinical trials for use against African sleeping sickness. Collectively, these findings suggest that the use of drugs targeting TRYS, in combination with existing drugs, could prove an effective strategy in treating *T. brucei* infections.

## Experimental procedures

### Cell lines and culture conditions

*Trypanosoma brucei* bloodstream-form ‘single marker’ S427 (*T7RPOL TETR NEO*) and knockouts were cultured at 37°C in modified HMI9 medium (56 μM 1-thioglycerol was substituted for 200 μM 2-mercaptoethanol) supplemented with 2.5 μg ml^−1^ G418 to maintain expression of T7 RNA polymerase and the tetracycline repressor protein (Hirumi and Hirumi, 1989). Cultures were initiated with 1 × 10^5^ cells ml^−1^ and subcultured when cell densities approached 1–2 (× 10^6^) ml^−1^. Single marker (WT), SKO, cDKO and OE cell lines were also grown continuously in the presence of the appropriate drug selection (see below for further details).

In order to examine the effects of inhibitors on the growth of these parasites, triplicate cultures containing the inhibitor were seeded at 1 × 10^5^ trypanosomes ml^−1^. Cell densities were determined using the CASY Model TT cell counter (Schärfe) after culture for 72 h. EC_50_ values were determined using the following two-parameter equation by non-linear regression using GraFit:


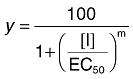


where the experimental data were corrected for background cell density and expressed as a percentage of the uninhibited control cell density. In this equation [I] represents inhibitor concentration and m is the slope factor.

### Generation of knockout, overexpression and recovery constructs

The primers used are summarized in Table 1 and were designed using the *T. brucei* trypanothione synthetase (*TRYS*) sequence in GeneDB (Tb927.2.4370) as a template. The accuracy of all assembled constructs was verified by sequencing. *TRYS* gene replacement cassettes were generated by amplifying a region of DNA encompassing the 5′-UTR, open reading frame (ORF) and 3′-UTR of *T. brucei TRYS* from genomic DNA with primers 5′UTR-NotI_s and 3′UTR-NotI_as, using *Pfu* polymerase. This sequence was then used as a template for the amplification of the individual regions used in the assembly of replacement cassettes containing the selectable drug-resistance genes puromycin *N*-acetyl transferase (*PAC*) and hygromycin phosphotransferase (*HYG*), exactly as previously described (Martin and Smith, 2005).

**Table 1:** Upper case letters refer to nucleotides corresponding to gene sequences in *T. brucei*; lower case refers to additional sequences used in generating constructs.

Primer name	Primer sequence
5′UTR-NotI_s	5′-ataagaatgcggccgcTGTGGTTGTTGTTTC-3′
5′UTR-HindIII/PmeI_as	5′-gtttaaacttacggaccgtcaagcttTTGTTTCAATTGCTTTTCC-3′
3′UTR-PmeI/BamHI_s	5′-gacggtccgtaagtttaaacggatccCACAACCCTTCCGTTTTG-3′
3′UTR-NotI_as	5′-ataagtaagcggccgcGGAATAAACATAAAGC-3′
*TbTRYS*-HindIII_s	5′-cggaaggagaaagcttatgggcagcagccat-3′
*TbTRYS*-BamHI_as	5′-ggatccCTACATTTGAATACGTACGGGACCAAACGGAGATTCGAGCCCAGTGATGAGCTT-3′
*TbTRYS*(C57A) mut_s	5′-cttttctgcggtttcaaataccaa**gc**tgtagaattt-3′
*TbTRYS*(C57A) mut_as	5′-aaattctaca**gc**ttggtatttgaaaccgcagaaaag-3′

Restriction endonuclease sites are underlined and mutations used to generate a amidase-dead TRYS are indicated in bold.

To generate the recovery construct pLew100*_TbTRYS*, the *TRYS* ORF from *T. brucei* was amplified from genomic DNA using *TbTRYS*-HindIII sense and *TbTRYS*-BamHI antisense primers, and cloned into pCR-Blunt II TOPO (Invitrogen). The pCR-Blunt II-TOPO-*TbTRYS* construct was then digested with HindIII and BamHI and the resulting fragment ligated into the HindIII/BamHI cloning site of a modified pLew100 tetracycline-inducible expression vector (Wirtz *et al*., 1999) which contains blasticidin S transferase (*BSD*) as the selectable gene marker (kindly supplied by Dr Kirstee Martin). The resulting rescue vector was also used as a *TRYS* overexpression vector in single marker *T. brucei* bloodstream parasites.

An additional recovery construct was generated containing a mutated version of the *T. brucei TRYS* gene. Site-directed mutagenesis was carried out following the QuikChange® protocol (Stratagene) and using the KOD HotStart DNA polymerase (Novagen). Using the pLew100_*TbTRYS* construct as a template, a C to A mutation was introduced at position 57 of *T. brucei* TRYS was generated by PCR resulting in the modified construct – pLew100_*TbTRYS*(C57A).

### Generation of transgenic *T. brucei* cell lines

Knockout, recovery and overexpression constructs were prepared using QIAprep Miniprep Plasmid Kit (Qiagen). DNA was digested with NotI, ethanol precipitated and redissolved in sterile water at a final concentration of 1 μg μl^−1^. Trypanosomes were electroporated with 5 μg of DNA using reagents from the Human T cell Nucleofector kit as per manufacturers' instructions and using programme X-001 of the Nucleofector II electroporator (Amaxa, Cologne, Germany) (Burkard *et al*., 2007).

The cDKO of *TRYS* was generated by sequentially replacing the endogenous TRYS genes with drug-resistance genes. The first *TRYS* allele was replaced with the drug-resistance gene PAC resulting in a SKO cell line which was cultured in the continuous presence of 0.1 μg ml^−1^ puromycin. The SKO cells were then transformed with either the recovery vector pLew100_*TbTRYS* or the mutated version of this vector pLew100_*TbTRYS*(C57A) and cultured in the presence of 2.5 μg ml^−1^ blasticidin in addition to puromycin. Prior to removal of the remaining *TRY*S allele, expression of the recombinant TRYS proteins were induced by the addition of tetracycline (2 μg ml^−1^, 72 h) to the culture medium. A cDKO cell line was finally generated following replacement of the final *TRYS* allele with an HYG. The resulting cell line was cultured in the presence of hygromycin (4.0 μg ml^−1^) in addition to the previously described drugs. The TRYS overexpression cell line was generated by directly transforming single marker bloodstream parasites with the pLew100_*TbTRYS* vector and inducing recombinant protein expression by the addition of tetracycline. At each step in the generation of these transgenic cell lines, parasites were cloned by serial dilution.

### Southern blot analyses of transgenic *T. brucei* cell lines

The ORF of *T. brucei TRYS* was amplified by PCR (using the primers previously described for the cloning of *Tb*TRYS into pLew100-*BSD*) and the PCR DIG Probe Synthesis Kit (Roche). The resulting digoxigenin-labelled *TbTRYS* product was used as a probe. Samples of genomic DNA (5 μg) from single marker and transgenic cell lines were digested with the restriction endonuclease PstI, the digestion products were then separated on a 0.8% agarose gel and transferred on to a positively charged nylon membrane (Roche). The membrane was hybridized overnight in DIG Easy Hyb solution (Roche) at 42°C with the DIG-labelled *TRYS* ORF probe (2 μl of PCR product). Following hybridization, membranes were washed twice in low stringency conditions (25°C, 2 × 5 min, 2× SSC with 0.1% SDS) and twice in high stringency conditions (68°C, 2 × 15 min, 0.5× SSC with 0.1% SDS), where 1× SSC comprises 150 mM sodium chloride and 50 mM sodium acetate, pH 7.0. Bound probe was detected using the DIG immunological detection kit (Roche) as per manufacturers' instructions.

### Western blot analysis of *T. brucei* cell lysates

Polyclonal antisera against *T. brucei* TRYS were raised in adult male Wistar rats. An initial injection of 100 μg of purified antigen, emulsified in complete Freund's adjuvant, was followed by two identical booster injections of antigen emulsified in Freund's incomplete adjuvant at 2 week intervals.

*Trypanosoma brucei* whole cell extracts (1 × 10^7^ parasites per lane) were separated by SDS/PAGE and subsequently transferred onto nitrocellulose. After blocking with 7% skimmed milk in phosphate buffered saline (PBS) for 1 h, blots were incubated with either *T. brucei* TRYS (1:700 dilution) or *T. brucei* PTR1 (1:500) (Sienkiewicz *et al*., 2008) polyclonal antiserum for 1 h, washed in PBS containing 0.1% (v/v) Tween-20, and then incubated with a secondary antibody [rabbit anti(rat IgG)] (Dako, Ely, UK; 1:10 000 dilution). Immunoblots were developed using the ECL plus (enhanced chemiluminescence) system from GE Healthcare (Piscataway, NJ, USA).

### Drug discovery

A high-throughput screening campaign of 63 000 compounds led to the identification of several novel chemical classes of *T. brucei* TRYS inhibitor. Chemical development of one of these lead compounds led to the identification of compound DDU 86439 (*N-*(3-(dimethylamino)propyl)-2-(3-(3-fluorophenyl)-1*H*-indazol-1-yl)acetamide) (patent pending) which inhibited recombinant TRYS with a potency of 0.045 μM and inhibited the growth of bloodstream trypanosomes with an EC_50_ of 6.9 ± 0.2 μM. DDU 86439 was prepared in five synthetic steps from indazole. Briefly, indazole was iodinated at the three position, as previously described (Edwards *et al*., 2003). The resultant aryl iodide served as the substrate for a Suzuki coupling reaction with 3-fluorophenylboronic acid in an analogous manner to that previously described (El Kazzouli *et al*., 2005). The indazole was then *N*^1^-alkylated by treatment with sodium hydride and ethyl bromoacetate. The resulting crude ester was hydrolysed by treatment with sodium hydroxide in water/tetrahydrofuran. Following acidic workup, the product carboxylic acid was purified by silica column chromatography. The amide synthesis was accomplished via standard carbodiimide chemistry utilizing *N-*(3-Dimethylaminopropyl)-*N*′-ethylcarbodiimide, 1-Hydroxybenzotriazole, *N*,*N*-Diisopropylethylamine and 3-(dimethylamino)-1-propylamine to furnish the title compound, which was purified by silica column chromatography. Full details of chemical synthesis of this and other inhibitors are to be published elsewhere.

### Analysis of intracellular thiols

To determine the effect of inhibitor DDU 86439 on intracellular thiol levels, cultures containing 1 × 10^5^ bloodstream trypanosomes ml^−1^ were incubated with varying concentrations of inhibitor DDU 86439 equivalent to 0.5, 1.0, 1.5 and 2× the previously determined EC_50_ (6.9 ± 0.2 μm). Following incubation for 72 h, cells (1 × 10^8^) were collected by centrifugation (900 *g*, 10 min, 4°C), washed once in ice-cold PSG buffer (PBS, pH 8.0, 1.5% (w/v) glucose and 0.5 mg ml^−1^ BSA) and derivatised with monobromobimane, as described previously (Shim and Fairlamb, 1988). Acid-soluble thiols were separated by ion-paired, reverse phase HPLC on an ion-paired Ultrasphere C_18_ column using a Dionex Ultimate 3000 instrument fitted with a Dionex RF-2000 fluorometer.

### *In vivo* studies

Wild-type and transgenic bloodstream *T. brucei* parasites were cultured in the absence of selectable drugs (hygromycin, blasticidin, puromycin and G418) for 24 h prior to infection of mice. During this time, cDKO cells were grown in the presence or absence of 1 μg ml^−1^ tetracycline. These parasites were then used to infect groups of five mice (dosed with and without doxycycline respectively) by a single intraperitoneal injection of 10^4^ parasites in 0.2 ml of HMI9-T medium. The plus doxycycline group of animals were dosed with doxycycline in their drinking water (0.2 mg ml^−1^ in a 5% sucrose solution) for 5 days prior to infection and freshly prepared every second day for the duration of the experiment. Animals were inspected daily for clinical signs of infection and wet smears of tail blood were examined microscopically. Parasitaemia was determined using a Neubauer haemocytometer, as previously described (Sienkiewicz *et al*., 2008). Mice that exceeded a parasitaemia > 10^8^ ml^−1^ were humanely killed, because prior experience indicated that animals would succumb to an overwhelming infection by the following day.
