# Supplementary material for: Dissecting the essentiality of the bifunctional trypanothione synthetase-amidase in Trypanosoma brucei using chemical and genetic methods
Source: Mol Microbiol. 2009 Jun 24;74(3):529–40. doi: 10.1111/j.1365-2958.2009.06761.x (PMC2784880; doi:10.1111/j.1365-2958.2009.06761.x)
Supplement: Supplementary file 1 [file mmi0074-0529-SD1.pdf]

Supporting Information:

Dissecting the essentiality of the bifunctional trypanothione synthetase-amidase in *Trypanosoma brucei* using chemical and genetic methods

Susan Wyllie, Sandra L. Oza, Stephen Patterson, Daniel Spinks, Stephen Thompson and Alan H. Fairlamb

Table S1. Intracellular thiols of *T. brucei* transgenic cell lines Thiols were determined as described in Materials and Methods. Each value represents the mean of triplicate determinations.

Cell line	Thiols nmol (10^8 cells)$^{-1}$ GSH	Thiols nmol (10^8 cells)$^{-1}$ $T[SH]_2$
WT	0.47 ± 0.05	0.38 ± 0.02
SKO	0.42 ± 0.02	0.25 ± 0.01
OE	0.56 ± 0.06	0.96 ± 0.05
cDKO + tetracycline	0.51 ± 0.06	0.42 ± 0.04

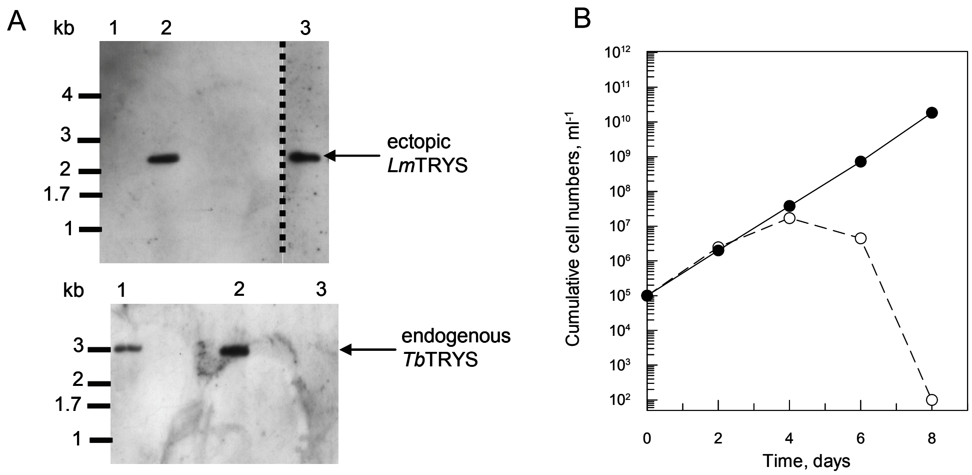

Fig. S1. Complementation of cDKO cells with ectopic *L. major TRYS.* (A) Confirmation of genotype of *T. brucei* TRYS conditional double knockout cell line. Southern-blot analysis of *Pst*I -digested genomic DNA (~5 μg) from wild-type *T. brucei* cells (lane 1), $LmTRYS^{Ti}\Delta TRYS$::PAC (lane 2) and $LmTRYS^{Ti}\Delta TRYS$::PAC/$\Delta TRYS$::HYG (lane 3). The *LmTRYS* ORF was used as a probe in the upper panel and shows the ectopic copy of $LmTRYS^{Ti}$ at ~2.5 kb. The *TbTRYS* ORF was used as a probe in the lower panel and the allelic TbTRYS can be seen at ~3 kb. Dotted line indicates where the southern image was cropped for clarity. **(B)** The growth of the cDKO cell line expressing *L. major* TRYS in HMI9-T media was monitored in the presence (closed circles) and absence of tetracycline (open circles, dashed line).

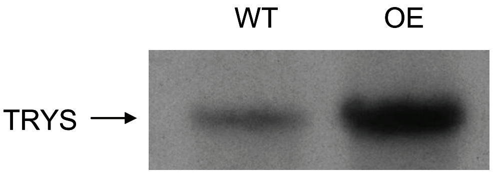

Fig. S2. Immunoblot analysis of a TRYS overexpressing cell line. Immunoblots of cell extracts of WT and OE (plus tetracycline) cells were probed with anti-serum to *T. brucei* TRYS (1 x 10^7 parasites in each lane).

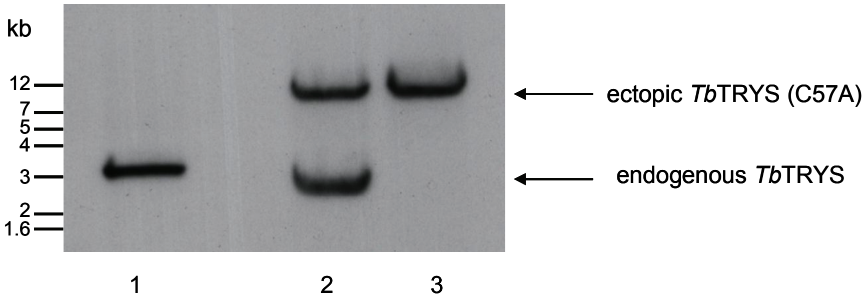

Fig. S3. Genotypic analysis of cDKO(C57A) cell line. Genotypic confirmation of the TRYS cDKO(C57A) cell line. Southern-blot analysis of PstI-digested genomic DNA (~5 μg) from wild-type *T. brucei* cells (lane 1), *TRYS(C57A)*^{Ti}Δ*TRYS*::PAC (lane 2) and *TRYS(C57A)*^{Ti}Δ*TRYS*::PAC/Δ*TRYS*::HYG (lane 3) cells; the *TRYS* ORF probe shows allelic *TbTRYS* at 3 kb and the ectopic copy *TbTRYS(C57A)*^{Ti} at ~10 kb.
